# Supplementary material for: Vitamin D Analogs Bearing C-20 Modifications Stabilize the Agonistic Conformation of Non-Responsive Vitamin D Receptor Variants
Source: Int J Mol Sci. 2022 Jul 30;23(15):8445. doi: 10.3390/ijms23158445 (PMC9369186; doi:10.3390/ijms23158445)
Supplement: Supplementary file 1 [file ijms-23-08445-s001.zip › ijms-1830127-supplementary.pdf]

Supporting information

Vitamin D analogs bearing C-20 modifications stabilize the agonistic conformation of non-responsive vitamin D receptor variants

Anna Y. Belorusova [1,2,3,4,†,‡], Daniela Rovito [1,2,3,4,5,‡], Yassmine Chebaro [1,2,3,4], Stefanie Doms [6], Lieve Verlinden [6], Annemieke Verstuyf [6], Daniel Metzger [1,2,3,4,5], Natacha Rochel [1,2,3,4,*] and Gilles Laverny [1,2,3,4,5,*]

1 Institut de Génétique et de Biologie Moléculaire et Cellulaire (IGBMC), F-67400 Illkirch, France;
2 Centre National de la Recherche Scientifique (CNRS), UMR 7104, F-67400 Illkirch, France;
3 Institut National de la Santé et de la Recherche Médicale (INSERM), UMR-S 1258, F-67400 Illkirch, France;
4 Université de Strasbourg, IGBMC UMR 7104 UMR-S 1258, F-67400 Illkirch, France;
5 OSCAR, French Network for Rare Bone Diseases
6 Clinical and Experimental Endocrinology, Department of Chronic Diseases and Metabolism, KU Leuven, 3000 Leuven, Belgium;
* Correspondence: rochel@igbmc.fr (N.R.); laverny@igbmc.fr (G.L.)
† Current address: Zobio B.V., Department of Assay Development and Screening, Leiden, 2333 CH Leiden, The Netherlands
‡ Joint first authors.

Content of SI:

X-ray crystal structure of BXL-62 bound to zVDR LBD. (Figure S1)

Schematic representation of the interactions of BXL-62 with zVDR or with zVDRgem. (Figure S2)

Close-up view around Leu(His)337. (Figure S3)

Distances between C20 atom of the ligand and Cγ of H333 and H423. (Figure S4)

Distances between C21 and C21a atoms of BXL-62 and Cγ of His333 and His423. (Figure S5)

Data collection and refinement statistics. (Table S1)

Protein - ligand aliphatic side chain distances (in Å) observed in the 1,25D, BXL-62 and Gemini-72 zVDR WT LBD complexes. (Table S2)

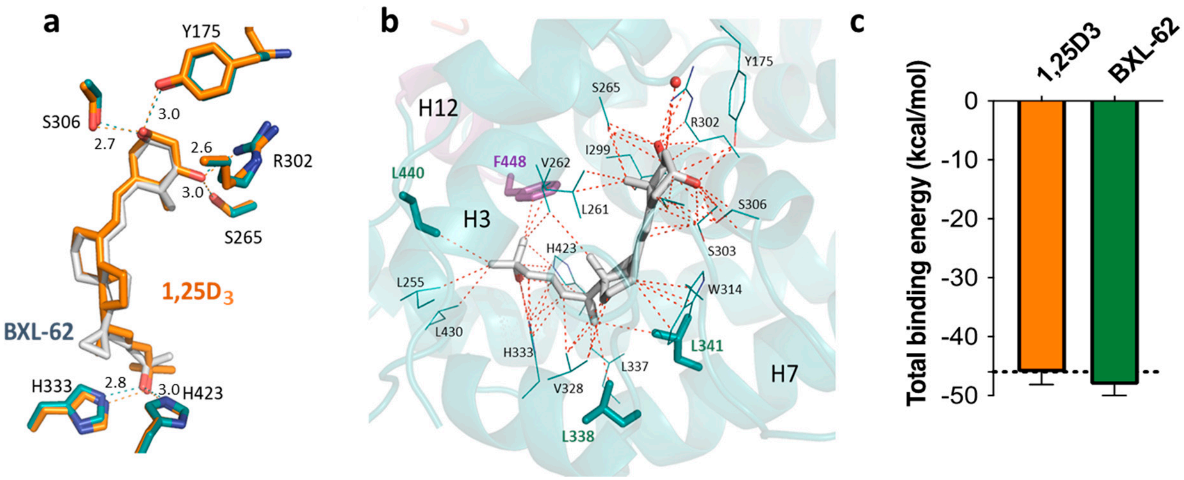

Figure S1: X-ray crystal structure of BXL-62 bound to zVDR LBD. a) Superposition of BXL-62 (gray) and 1,25D$_3$ (orange). Hydrogen bonds are shown by dotted lines. Distances in Å for hydrogen bonds formed by BXL-62. **b**) Interactions of BXL-62 with VDR residues within 4 Å distance are shown by red dotted lines. Residues that form stronger interactions with BXL-62 than with 1,25D$_3$ are shown in thicker. **c)** Total binding affinities for BXL-62 and 1,25D$_3$.

zVDR – BXL-62 zVDRgem – BXL-62

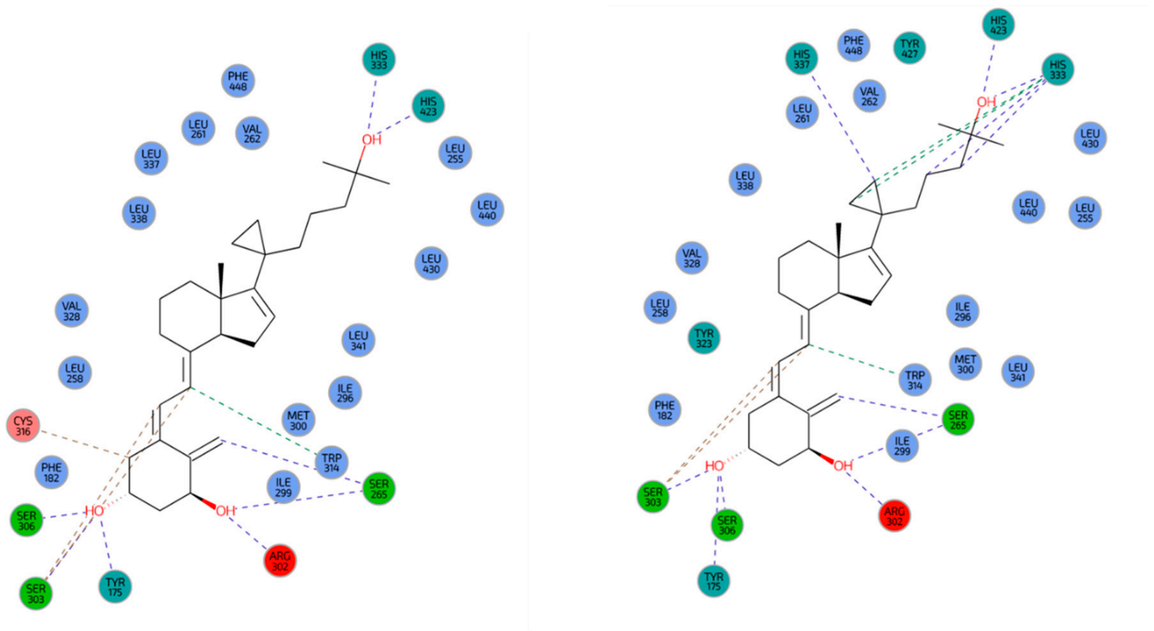

Figure S2. Schematic representation of the interactions of BXL-62 with zVDR, or with zVDRgem. Interactions of BXL-62 with zVDR or zVDRgem residues within 4 Å distance are shown by dotted lines. Color code: blue, hydrophobic residue; cyan, aromatic residue; pink, cysteine; red, positively charged residue; green, polar residues

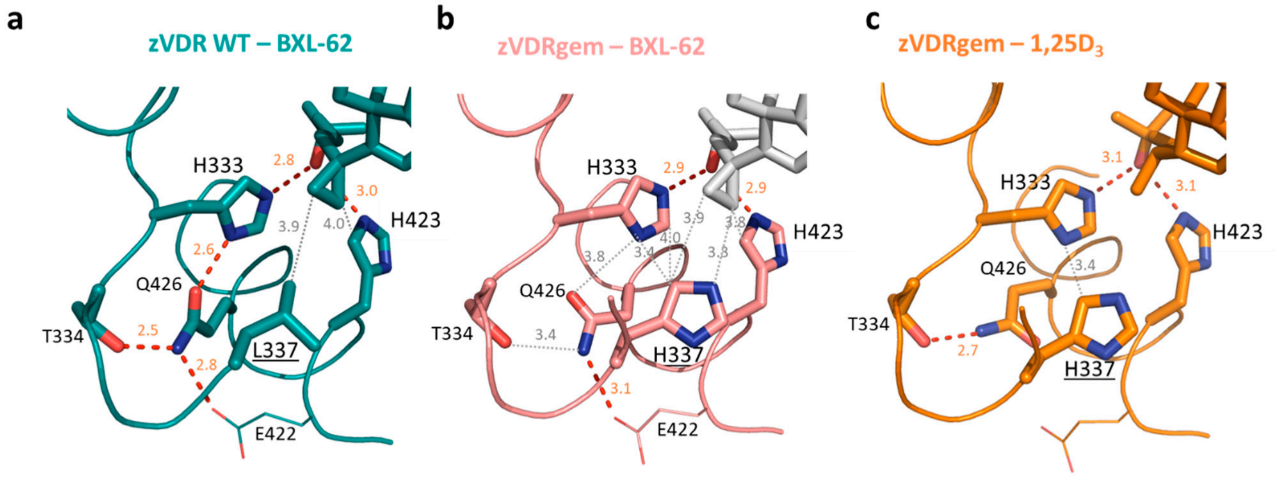

Figure S3: Close-up view around Leu(His)337 in the zVDR WT - BXL-62 (**a**), zVDRgem - BXL-62 (**b**) and zVDRgem – 1,25D_3 (**c**). Grey dashed lines correspond to ligand interactions with VDR residues within 4 Å distance and red dashed lines to hydrogen bonds. Distances are in Å.

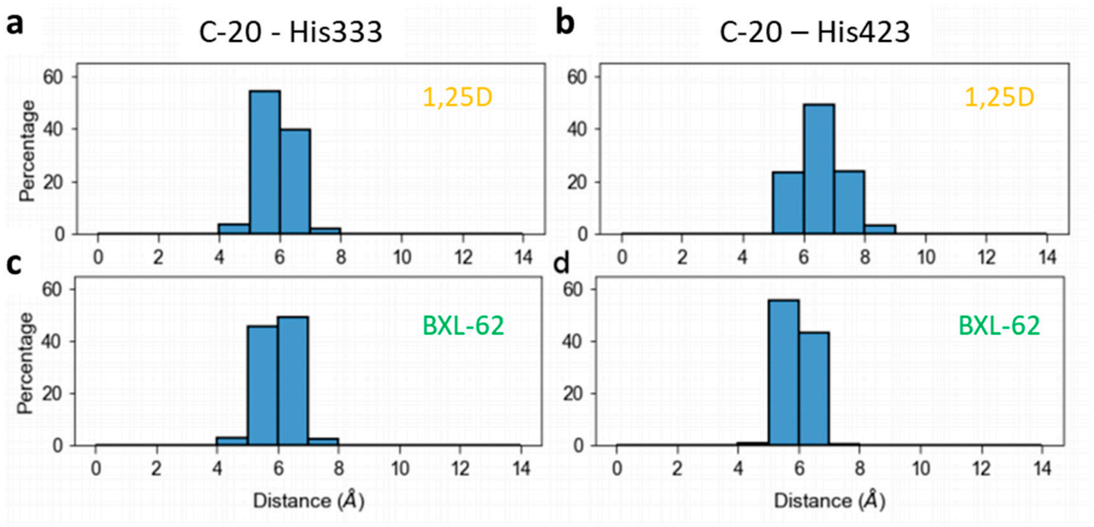

Figure S4: Distances between the Cγ of H333 and H423 of VDRgem and the C20 atom of $1,25D_3$ (**a,b**) and of BXL-62 (**c,d**).

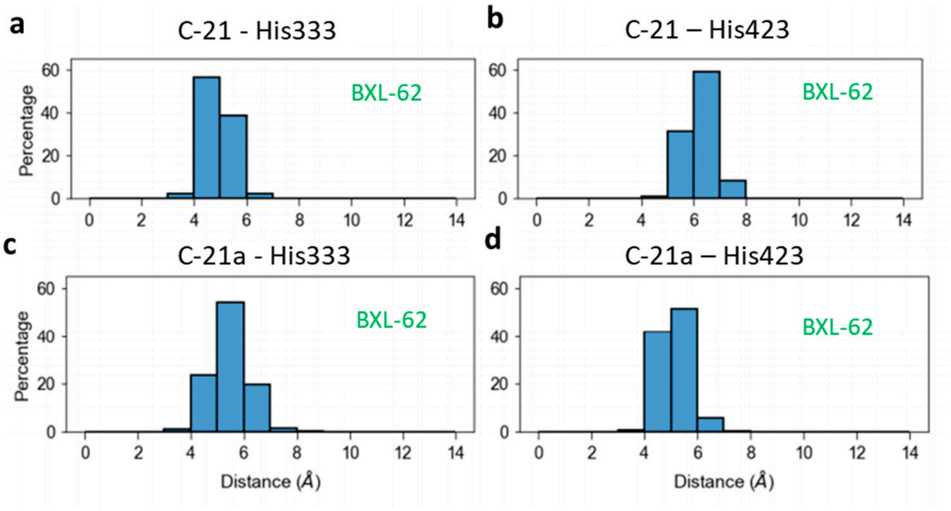

Figure S5: Distances between the Cγ of H333 and H423 of VDRgem and the C21 and C21a atoms of $1,25D_3$ (**a,b**) and of BXL-62 (**c,d**).

Table S1: Data collection and refinement statistics.

	zVDR WT - BXL-62	zVDRgem - BXL-62
**PDB ID**	**7BNS**	**7BNU**
**Data Processing**		
X-ray source	ID29	ID23-2
λ	0.98 Å	1.00 Å
Temperature	100 K	100k
Resolution (Å)	24.8-2.7	29.4-2.4
Crystal space group	$P6_522$	$P6_522$
Cell parameters (Å)	a=b=65.87; c=263.68	a=b=65.66 ; c=264.14
Unique reflections	9994	14068
Mean redundancy	1.3	2.0
Completeness (%)	98.38	99.17
Mean I/σ	9.4	12.25
$CC_{1/2}$	1	1
**Refinement**		
Resolution (Å)	24.8-2.7	29.4-2.4
Number of non-hydrogen atoms		
Protein	2002	1988
Ligand	31	31
Water molecules	38	72
Rcryst (%)	17.94	19.0
Rfree (%)	26.14	26.1
RMSD bond length (Å)	0.007	0.006
RMSD bond angles (˚)	0.84	0.76
Ramachandran plot (%)		
Core	97.52	97.92
Allow	2.48	2.08

Table S2. Protein - ligand aliphatic side chain distances (in Å) observed in the 1,25D$_3$, BXL-62 and Gemini-72 zVDR WT LBD complexes excluding the interactions with the hydroxyl groups. A cutoff of 4.0 Å has been used. For comparison purposes, contacts with 1,25D$_3$ greater than 4.0 Å are indicated in italics.

		**zVDR WT**			**zVDRgem**
**Residue**	**Atom**	**1,25D$_3$**	**Gemini-72**	**BXL-62**	**BXL-62**
		PDB: 2HC4	PDB: 3O1D	PDB: 7BNS	PDB: 7BNU
zLeu255(hLeu227) H3	CD1	C26(3.8)	F4(3.6),F5(3.6),C32(3.9)	C26(3.9)	C26(4.0)
zVal262(hVal234) H3	CG1	*C24(4.8)*	F2(3.7),F1(3.8)	C27(3.9)	C27(4.0)
	CG2	C24(3.7)	F2(3.3)	C27(3.7)	C27(4.0)
zIle296(hIle268) H5	CD1	*> 5*	C22(3.8)		
zMet300(hMet272) H5	CE	*C21(4.8)*	C24(3.5),C26(3.6)		
zVal328(hVal300) H6	CG1	C21(4.0)		C21(3.7),C22(3.9)	C21(3.8),C22(4.1)
zAla331(hAla303) H6	O	*C26(4.3)*	F6(3.3)		
zHis333(hHis305) L6-7	NE2	C23(3.7),C26(3.7),	F6(3.2),C28(3.7)	C22(3.8),C23(3.4)	C22(3.8),C23(3.5),
	CD2	C24(4.1)	C29(3.5)	C24(3.4)	C24(3.4)
	CE1	C23(3.9),C21(4.0)	C23(4.0),C24(3.8)	C22(3.6),C24(3.9)	C22(3.8),C24(4.1)
zLeu337(hLeu309) H7	CD2	C21(3.7)	C27(4.0)	C21a(3.8)	C21a(3.8)
zHis337 H7	CD2				C21a (3.8)
	NE2				C21a(3.3)
zLeu338(hIle310) H7	CD2	*C21(4.6)*	C27(3.8)	C21(3.8)	C21(3.9)
zLeu341(hLeu313) H7	CD2	*C21(4.4)*	C27(3.5)	C21(3.9)	C21(3.5)
zLeu419(hLeu393) H10	O	*>5*	C26(3.5)		
zHis423(hHis397) H11	NE2	C24(3.9)	F1(3.1),C30(4.0)	C27(3.8)	C27(3.7)
	CD2	*C21(4.3)*	C22(3.9),C24(3.7)	C21a(3.8)	C21a(3.8)
zLeu430(hLeu404) H11	CD2	C26(3.8)	F5(3.3),F6(3.3), C32(3.9)	C26(3.7)	C26(4.0)
zLeu440(hLeu414)L11-12	CD2	*C27(4.3)*	F5(3.6)	C26(4.0)	C26(4.1)
zVal444(hVal418) H12	CG1	C27(4.0)	F3(3.7)		
zPhe448(hPhe422) H12	CE2	*C26(4.4)*	F1(3.4)	C27(3.8)	C27(3.8)
